# A Rare Case of Diffuse Idiopathic Pulmonary Neuroendocrine Cell Hyperplasia

**DOI:** 10.1155/2015/318175

**Published:** 2015-11-02

**Authors:** Godwin Ofikwu, Vishnu R. Mani, Ajai Rajabalan, Albert Adu, Leaque Ahmed, Dennis Vega

**Affiliations:** ^1^Department of Surgery, Columbia University College of Physicians and Surgeons at Harlem Hospital, New York, NY 10037, USA; ^2^Department of Surgery, New York University School of Medicine, New York, NY 10016, USA; ^3^Department of Cardiothoracic Surgery, Harlem Hospital Center, New York, NY 10037, USA

## Abstract

Diffuse idiopathic pulmonary neuroendocrine cell hyperplasia (DIPNECH) is a rare clinical condition with only about 100 cases reported in the literature. It is characterized by primary hyperplasia of pulmonary neuroendocrine cells (PNECs) which are specialized epithelial cells located throughout the entire respiratory tract, from the trachea to the terminal airways. DIPNECH appears in various forms that include diffuse proliferation of scattered neuroendocrine cells, small nodules, or a linear proliferation. It is usually seen in middle-aged, nonsmoking women with symptoms of cough, dyspnea, and wheezing. We present a 45-year-old, nonsmoking woman who presented with symptoms of DIPNECH associated with bilateral pulmonary nodules and left hilar adenopathy. Of interest, DIPNECH in our patient was associated with metastatic pulmonary carcinoids, papillary carcinoma of the left breast, oncocytoma and angiomyolipoma of her left kidney, and cortical nodules suggestive of tuberous sclerosis. She had video assisted thoracoscopic surgery (VATS), modified radical mastectomy with reconstruction, and radical nephrectomy. She is currently symptom-free most of the time with over two years of follow-up.

## 1. Introduction

Diffuse idiopathic pulmonary neuroendocrine cell hyperplasia (DIPNECH) is a relatively new and rare disease that has recently received attention, and it is commonly misdiagnosed. DIPNECH was first reported by Aguayo et al. [1] in 1992. The World Health Organization (WHO) included DIPNECH in its 1999 lung tumor classification as a form of preinvasive lung lesion within the spectrum of neuroendocrine cell neoplasia [2]. This included tumorlets that extended beyond the basement membrane and formed lesions which are <5 mm in greatest dimension and nodules which are >5 mm. These were classified as pulmonary neuroendocrine tumors [NETs] and also known as carcinoids. DIPNECH is regarded as a precursor lesion for carcinoid tumors. Carcinoid tumors are subdivided into typical carcinoids, which are low grade tumors and atypical carcinoids, which are intermediate grade tumors. DIPNECH has a predilection for nonsmoking, middle-aged women and is associated with a predominantly obstructive ventilatory pattern on pulmonary function tests seen in obliterative bronchiolar fibrosis [1] or bronchiectasis. Most of the patients present with nonproductive cough and exertional dyspnea that are commonly misdiagnosed for bronchial asthma or chronic bronchitis. The treatments included systemic and inhaled corticosteroids, bronchodilators, and lung resection. The clinical outcomes vary from improvement of symptoms to deterioration. We present our experience in the management of a unique case of a 45-year-old, nonsmoking woman who presented with bilateral pulmonary nodules and left hilar adenopathy in association with papillary carcinoma of the left breast, oncocytoma, and angiomyolipoma of the left kidney. She gave a history of several years of a nonproductive cough, dyspnea, and wheezing that were initially thought to be due to bronchial asthma. The patient was managed with video assisted thoracoscopic surgery (VATS) with wedge resections for DIPNECH, left modified radical mastectomy for breast cancer, and left laparoscopic radical nephrectomy for oncocytoma. Her symptoms have progressively improved following the lung surgery, and she is currently asymptomatic most of the time. This case is unique because, to the best of our knowledge, it is the first case of DIPNECH with metastatic carcinoid associated with papillary breast cancer, ductal carcinoma in situ (DCIS), oncocytoma, and angiomyolipoma of the kidney with possible brain tumors. There may be an underlying genetic predisposition to development of these preinvasive lesions and neoplasms.

## 2. Case Summary

Our patient is a 46-year-old, nonsmoker, African American woman, who is originally from Barbados. She initially went to her primary care physician (PCP) four years prior to presenting to us, with several years history of cough, dyspnea, wheezing, and occasional chest pain. She was diagnosed with allergic rhinitis and asthma with a peak expiratory flow rate (PEFR) of 145 and 180 before and after albuterol treatment, respectively. The patient was treated with albuterol inhaler and antihistamines and continued follow-up visits to her PCP. The purified protein derivative (PPD) skin test was found to be positive. However, the follow-up chest X-ray was unremarkable. She was later called back to her PCP office for an abnormal result on her annual screening mammography. Additional views were required and were subsequently reported as normal, other than the three benign cysts that were found in her right breast.

A year later, she had an emergency room visit for an acute exacerbation of her respiratory symptoms. Due to the recurrent emergency room visits, her PCP established a more aggressive asthma treatment plan. Several weeks later at her annual visit to the gynecologist, the patient was found to have a palpable mass on the upper outer quadrant of her left breast, measuring 2 × 3 cm. There was no associated axillary lymphadenopathy. A core needle biopsy of the left breast mass showed papillary carcinoma, estrogen receptor (ER) 90–95% positive, progesterone receptor (PR) 65–70% positive, Her2/Neu receptor (HER2) by IHC 1+ negative, and Ki-67 < 10% favorable. During preoperative breast MRI and mammogram, multiple grouped micro calcifications were seen in the right breast at 12 o'clock position, suspicious of malignancy, and grouped micro calcifications at 12 and 6 o'clock position in left breast. Stereotactic biopsies of these right breast grouped micro calcifications and left breast grouped micro calcifications at 12 o'clock position showed fibrocystic changes and ductal hyperplasia in the right breast and carcinoma in the left breast.

**Figure 1:**
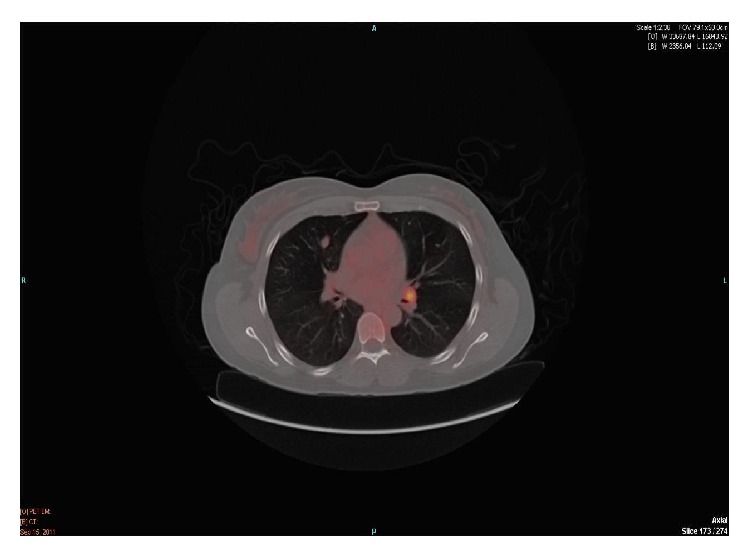
PET CT of chest.

**Figure 2:**
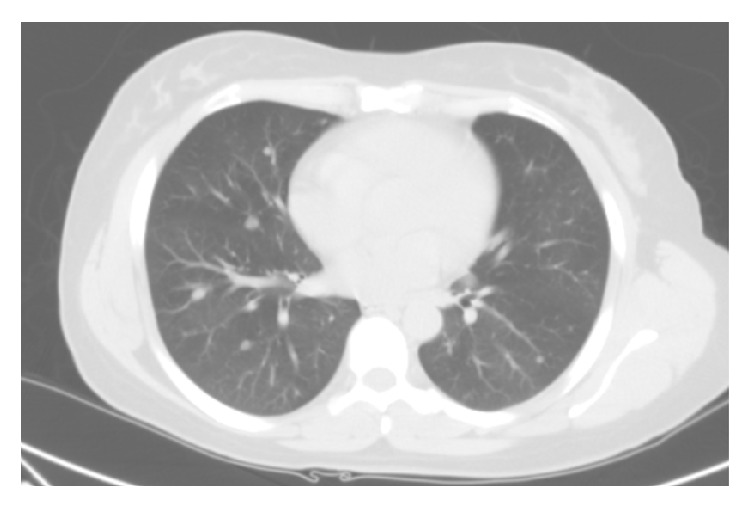
CT chest. Multiple subcentimeter bilateral lung nodules.

A CT scan of the abdomen and pelvis was done as part of the workup to determine the extent of her disease. It showed a 3.5 cm mass with central necrosis in the upper pole of the left kidney, a smaller exophytic lesion of the mid-portion of the left kidney, bone island, or area of sclerosis in left iliac wing, and the limited view of the lung bases demonstrated multiple subcentimeter nodules in the lung bases. Findings appeared consistent with primary renal neoplasm with lung nodules of unknown etiology versus metastatic renal carcinoma. A CT guided biopsy of the left renal mass showed oncocytic renal cell neoplasm, which was suggestive of oncocytoma. The CEA was 1.5 ng/mL (*n* < 5 ng/mL); CA-125, 3.7 (*n* < 35); and CA-19-9, 27.29 (*n* < 39.6). The subsequent chest CT (Figure 2) showed a triangular shaped soft tissue density in the anterior mediastinum and bilateral multiple pulmonary nodules with the largest nodule located in the right upper lobe, measuring 1.5 cm, left hilar adenopathy, and minimal focal pleural thickening in the left lower hemithorax. A Positron Emission Tomography (PET) scan imaging (Figure 1) showed uptake in these lung nodules as well as in the left hilar region and in the left renal mass. A CT guided transthoracic needle biopsy (TTNBx) of the largest right pulmonary nodule was nondiagnostic.

A multidisciplinary review of the PET/CT (Figure 4) raised the possibility that the lung lesion was metastatic renal cell or breast cancer versus unknown primary. It was decided that the best way to obtain a diagnosis was to operate and perform wedge resection of the largest lung nodules. She had bilateral video assisted thoracoscopic surgery (VATS) with resection of the right chest 2 cm hilar lymph node, wedge resections of right middle lobe (RML), and excision of right lower lobe (RLL) nodules. The patient did well postoperatively and was discharged home on postoperative day three. The pathology of the left hilar lymph node was positive for carcinoid tumor. The right middle and lower lobe nodules showed carcinoid tumor as well. Also seen within the lung were multiple carcinoid tumorlets, diffuse neuroendocrine cell hyperplasia, and bronchiectasis. Microscopically, the tumor in both the lungs and lymph nodes showed organoid and trabecular growth with salt and pepper chromatin. The highest mitotic count was 1 per 10 high power fields (HPF) and no necrosis was seen. These findings are consistent with typical carcinoid tumors. Given the history of breast cancer and a renal tumor, immunohistochemistry (IHC) studies were performed. IHC staining was positive for cytokeratin (weak), cytokeratin 7, synaptophysin, chromogranin, and TTF1 (weak) but negative for napsin, PAX8, GFDFP15, ER, PR, mammaglobin, and CDX2.

The patient then underwent a left mastectomy and sentinel lymph node biopsy. The pathology on the sentinel lymph node was negative for malignancy. The left breast on pathology showed florid low grade multifocal DCIS. IHC studies were similar to her previous breast biopsy result with no focus on invasiveness of DCIS. She also had laparoscopic radical left nephrectomy for her left renal tumors. The pathology result returned as oncocytoma with two growth patterns. One was of solid growth with oncocytes and the other with tubular and nested growth of oncocytes. Oncocytes were positive for CK18, CK7, CD10, c-kit, racemase, and PAX8 and negative for vimentin, thyroid transcription factor 1 (TTF-1), napsin, chromogranin, and synaptophysin. The second tumor is spindle cell predominant, positive for SMA and HMB45 and negative for CK, representing an angiomyolipoma.

**Figure 3:**
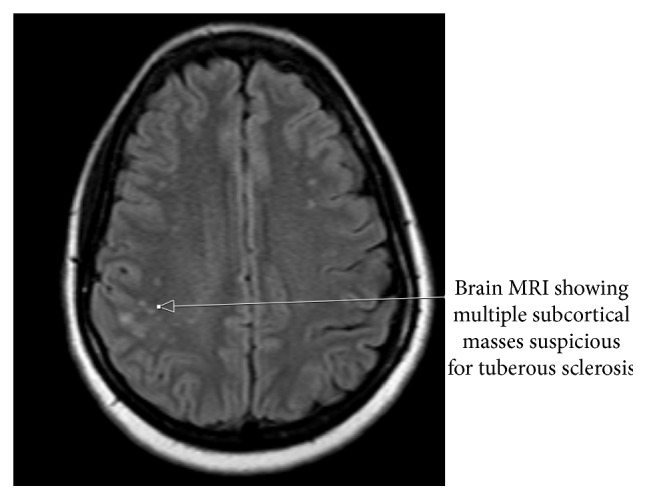
Brain MRI, showing subcortical masses.

**Figure 4:**
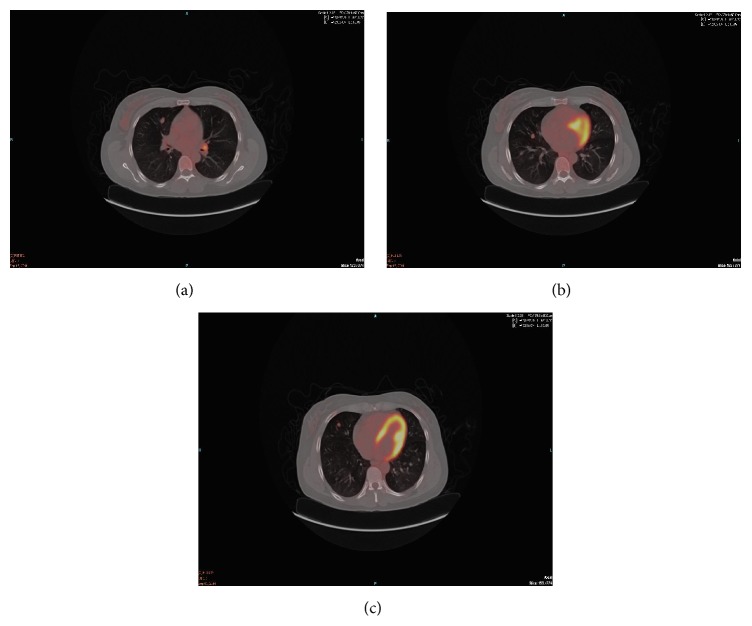
Multidisciplinary review of PET CT.

A brain MRI was later done because of the association of DIPNECH with MEN1 and tuberous sclerosis with angiomyolipoma of the kidney. MRI of the brain (Figure 3) showed flair hyperintense lesions in subcortical white matter of the right parietal lobe, which could represent subcortical tubers in the setting of Tuberous Sclerosis Complex (TSC). The patient then had a whole body octreotide scan, which, in addition to the masses discussed thus far, showed bilateral anterior neck masses. Fine needle aspiration biopsy (FNAB) was reported as benign. The patient refused further invasive testing to get a tissue diagnosis.

In summary, we have a patient with bilateral diffuse idiopathic pulmonary neuroendocrine hyperplasia (DIPNECH), lung carcinoid tumor with mediastinal metastasis and unrelated left breast cancer with florid multifocal DCIS, left renal tumors, anterior neck tumors, and possible tuberous sclerosis.

## 3. Discussion

The first formal recognition of DIPNECH was by Aguayo et al., in a series of 6 case reports in 1992, in which they described patients with severe obstructive airway disease and progressive dyspnea. These patients were discovered to have diffuse neuroendocrine cell hyperplasia of the airways and obliterative airway fibrosis, in which the three of these patients also had carcinoid tumor. Since DIPNECH was reported in 1992, several other authors have reviewed additional cases of DIPNECH; some were associated with carcinoids [1]. This recent surge in frequency of the diagnosis of DIPNECH is due to increased use of high resolution CT (HRCT) scans combined with increased awareness of this entity as previously suggested by Davis et al. [3]. It is noteworthy that only about 100 cases of DIPNECH have been reported in the literature. In 1999, DIPNECH was defined by the WHO as a generalized proliferation of scattered single cells, small nodules/neuroendocrine bodies, or linear proliferations of pulmonary neuroendocrine cells that may be confined to the bronchiolar epithelium, including local extraluminal proliferation in the form of tumorlets, or extend to the development of carcinoid tumors. This WHO definition includes cases of neuroendocrine cell hyperplasia and tumorlet formation with or without associated airway fibrosis and specifically notes that other pathologies that might be associated with reactive neuroendocrine cell proliferation are absent [4]. DIPNECH has been associated primarily with the development of typical carcinoids. In fact, it is generally believed that DIPNECH is probably a precursor lesion for typical carcinoid. WHO report indicated this in its 2004 histological classification of tumors. In the series by Davies et al., there were three cases associated with atypical carcinoid, one of whom had multiple endocrine neoplasia. To our knowledge, this is the first case of DIPNECH where the patient is presenting with a classic clinical picture of DIPNECH with metastatic carcinoid and multiple unrelated tumors. Our patient has DIPNECH in association with papillary carcinoma of the breast, oncocytoma, and angiomyolipoma of the left kidney and a yet-to-be-diagnosed neck mass.

Our patient is a nonsmoking woman who was first seen by a physician in the United States 4 years prior to presenting to us. The patient complained of many years of intermittent, nonproductive cough, dyspnea, wheezing, and occasional chest pain. There was no significant weight loss or physical findings. The patient appeared to be in good health, otherwise, and was initially misdiagnosed as case of bronchial asthma and treated for it. The patient was later found to have papillary breast cancer and renal tumors; she was again misdiagnosed as having metastatic lung disease when her chest CT revealed multiple subcentimeter round nodules in the lung bases. This presentation is in line with several cases discussed in the literature. A 6-year electronic literature search of all DIPNECH cases reported in English by Nassar et al. found that 92% of these patients were women with an age range of 36 to 76 years at diagnosis and 67% of them were nonsmokers. This description applies to our patient. Furthermore, the latter report also found that 71% of the patients had cough; 63% had dyspnea; and 25% had wheezing for days to years prior to diagnosis. Like our patient, 54% of the patients reviewed had pulmonary function tests showing obstructive ventilatory disease and pulmonary nodules were found in 63% of patients. Grozinsky-Glasberg et al., in their review of all the 100 reported cases of DIPNECH identified in the literature published in 2011, found a similar pattern of clinical presentation and most of the patients had stable disease like our patient who has stable symptoms that have not progressed since diagnosis. In fact, her symptoms are so stable that she does not want to undergo further evaluation for a recently discovered neck mass since fine needle aspiration returned as benign cells.

After reviewing the PET/CT of our patient, the differential diagnosis included metastatic renal cell cancer; metastatic breast cancer, although unlikely given in this picture; and metastatic disease of unknown primary. Several reviews in the literature have alluded to the fact that most DIPECH cases are usually misdiagnosed and these patients often get treated for bronchial asthma and other respiratory conditions. When pulmonary nodules are encountered in the lungs they are believed to be metastases. A surgical lung biopsy was required to make a histological diagnosis of DIPNECH and carcinoids with bronchiectasis in this patient. 21% of patients reported in the literature, as reviewed by Nassar et al., had associated bronchiectasis with DIPNECH and 88% of them required surgical lung biopsy for histological confirmation of disease.

After the histological diagnosis of DIPNECH with metastatic carcinoids, we thought that the other masses including the breast and kidney masses were metastatic carcinoids. However, immunohistochemical and histological studies, as indicated above, showed the breast mass to be a papillary carcinoma which is ER+, PR+ HER2/NEU−, and Ki67−. The renal masses were negative for TTF-1, napsin, chromogranin, and synaptophysin and had no histological appearance of carcinoids. In contrast, the neuroendocrine cells in DIPNECH are positive for neuroendocrine markers such as synaptophysin, chromogranin, and CD56. Davies et al. also showed weak positive staining for TTF-1 in nine cases [3]. These findings led us to conclude that the breast and renal masses were not metastatic carcinoids but were different and separate tumors. Incidence of multiple cancers in this patient led us to hypothesize that she might have familial or genetic predisposition to the occurrence of such a conglomerate of different tumors with benign, preinvasive, and malignant components. The patient denied any family history or similar symptoms in her immediate or extended family. However, we cannot rule out the presence of similar but subclinical conditions in her family. It is of interest that she is the only one in her family residing in the United States with every other family member back in Barbados and unavailable for examination and possible genetic testing.

The management of patients with DIPNECH is predominantly close surveillance. The role of surgery is not clearly defined. However, the treatment principle remains surgical intervention for early disease and predominantly palliative therapy in metastatic disease. Treatment strategies include systemic and inhaled corticosteroids, bronchodilators, and lung resection. Of note, the recent demonstration of molecular signaling pathways involved in tumor growth, as demonstrated by Chen et al. [5], showing that carcinoid and other neuroendocrine tumors have activation of Ras signaling directly by mutations in Ras and indirectly by loss of Ras-regulatory proteins, or via constitutive activation of upstream or downstream effector pathways of Ras, such as growth factor receptors or PI3-Kinase and Raf/MAP kinases might lead to new immunomodulating agents for the disease. In addition, aberrant activation of Ras signaling sensitizes cells to apoptosis when the activity of the PKC*δ* isozyme is suppressed and that PKC*δ* suppression is not toxic to cells with normal levels of RAS p21 signaling. Furthermore, inhibition of PKC*δ* by a number of independent means, including genetic mechanisms (shRNA) or small molecule inhibitors, is able to efficiently and selectively repress the growth of human neuroendocrine cell lines derived from bronchopulmonary, foregut, or hindgut tumors. PKC*δ* inhibition in these tumors also efficiently induced apoptosis. In line with these findings and more recent reports, Sunitinib, a multitargeted tyrosine kinase inhibitor, has been shown to improve the survival of pancreatic NETs patients. Similarly, the use of an mTOR inhibitor, everolimus, either alone or in combination with somatostatin analogs has demonstrated encouraging efficacy in treating advanced NETs as documented by Leung et al. [6]. The success of these two agents in pancreatic NETS supports the notion that targeting angiogenesis and/or PI3K/AKT/mTOR pathway is an important strategy for making therapeutic advances in this disease. The major drawback is the differential response to biological agents amongst NETs of different anatomical origins, with pancreatic NETs generally more responsive to both chemotherapy and targeted agents than NETs of other sites. There are currently many ongoing trials in exploring the role of other biological modifiers or agents in treating patients with NETs or carcinoids, especially metastatic disease. From the time our patient was first diagnosed, she has been treated with bronchodilators and inhaled steroids. Because of her unique presentation, she has had left modified radical mastectomy, left nephrectomy and bilateral VATS with removal of left chest 2 cm hilar lymph node, and right chest wedge resections of right middle lobe (RML) and right lower lobe (RLL) nodules for the lesions described above. She did well and was discharged home on postoperative day three. She has been followed up in clinic over a 2-year period postoperatively and reported to be asymptomatic most of the time, developing cough only when exposed to cold environment. This contributed to her refusal for any further evaluation and treatment of her condition. Her stable condition compliments the findings of Swigris and colleagues who presented four patients with 2- to 14-year follow-up with an excellent long-term prognosis. We anticipate an excellent long-term prognosis for our patient too.

## 4. Conclusion

There is a surge in the number of patients diagnosed with DIPNECH since it was first described in 1992. Patients with this unique disease are still misdiagnosed. Although most patients enjoy a relatively stable clinical course, this disease may be associated with prolonged morbidity in some patients. High index of suspicion and close follow-up remain the key for early diagnosis and management of patients with morbid symptoms.
